# Molecular Detection of *Coxiella burnetii*, *Rickettsia africae* and *Anaplasma* Species in Ticks from Domestic Animals in Lesotho

**DOI:** 10.3390/pathogens10091186

**Published:** 2021-09-14

**Authors:** Sibonginhlanhla I. C. Mahlobo-Shwabede, Oliver T. Zishiri, Oriel M. M. Thekisoe, Mabusetsa J. R. Makalo

**Affiliations:** 1 Discipline of Genetics, College of Agriculture, Engineering and Science, University of KwaZulu-Natal, Durban 4000, South Africa; Zishiri@ukzn.ac.za; 2 Unit for Environmental Sciences and Management, North-West University, Potchefstroom 2531, South Africa; oriel.thekisoe@nwu.ac.za; 3 Department of Livestock Services, Ministry of Agriculture and Food Security, Maseru 100, Lesotho; mabusetsa1930@gmail.com

**Keywords:** *Anaplasma* species, *Coxiella burnetii*, Lesotho, *Rickettsia africae*, ticks, coinfection, zoonoses

## Abstract

Tick-borne diseases (TBDs) hamper the growth of the livestock sector and impose major constraints for the health and management of domestic animals in the tropic and subtropical regions globally. Currently, there is no scientific report on the presence of zoonotic pathogens transmitted by tick species in Lesotho. This study aimed to identify zoonotic tick-borne pathogens of economic importance from ticks infesting domestic animals in Lesotho using molecular techniques. A total of 322 tick DNA pools were subjected to PCR screening for the presence of zoonotic pathogens and sequenced. The overall prevalence of *Anaplasma* spp. was 35% (113/322), with a 100% infection rate in *Rhipicephalus microplus*, followed by *R*. *evertsi evertsi* (92%), *Hyalomma rufipes* and *Otobius megnini* sharing 50% and the lowest infection rate was observed in *R*. *decoloratus* with 40%. The prevalence of *Coxiella burnetii*, a gram-negative pleomorphic etiological agent of Query fever (Q fever), was 1% (2/322) for all screened samples, with 20% of *R*. *decoloratus* and 1% of *R*. *e*. *evertsi* infected. *Rickettsia africae* was detected from *Hyalomma rufipes* with a 70% prevalence. This study provides a baseline knowledge of tick-borne pathogens of medical and veterinary importance in Lesotho and raises awareness of the prevalence of such diseases within the tourism sector as they are mostly affected.

## 1. Introduction

Tick-borne diseases (TBDs) hamper the growth of the livestock sector and impose major constraints for the health and management of domestic animals in the tropic and subtropical regions globally [1,2]. Ticks are vectors of many diseases in animals and humans worldwide. Pathogens carried and transmitted by ticks include bacteria, protozoa and viruses of medical and veterinary importance [3]. Eighty percent of the world’s livestock population is affected by TBDs as they hinder livestock production globally, with severe effects being observed mostly in rural populations where livestock are an essential source of income and food supply [4]. South Africa (SA) is an agro-exporting nation depending mainly on livestock productivity for subsistence [4,5]. In sub-Saharan Africa, tick-borne zoonotic pathogens are endemic, including *Anaplasma* spp., *Coxiella burnetii*, *Rickettsia* spp. and *Ehrlichia* spp., and the information available is still of concern for these pathogens [6]. The occurrence of livestock as hosts for ticks in SA promotes the widespread occurrence of TBDs [7].

Anaplasmosis is an emerging vector-borne disease that affects domestic animals globally and is known to manifest as acute or non-clinical infection [8]. Granulocytic anaplasmosis (GA) is an infectious multi-organ human and animal disease. *Anaplasma phagocytophilum* is known as the aetiological agent of anaplasmosis in different host species, that is, humans (HGA agent—human granulocytic anaplasmosis), which is a zoonotic rickettsial infection of neutrophils transmitted by ticks [7,9], horses (*A*. *phagocytophilum*/*Ehrlichia equi*) and dogs (*A*. *phagocytophilum*) [10]. The aetiological agents are obligate intracellular gram-negative bacterial species [10]. *Anaplasma phagocytophilum* and *Ehrlichia ewingii* colonise host granulocytes, whilst *E*. *canis*, *E*. *ruminantium* and *E*. *chaffeensis* colonise mononuclear phagocytes [10]. Clinical anaplasmosis results in loss of milk production and weight loss, while high fever and anorexia are commonly observed in domestic animals [11]. *Anaplasma* spp., closely related to *A*. *phagocytophilum,* have been described in canine blood in SA [12]. Regardless of these reports on pathogens, no true incidence has been properly investigated in southern Africa. 

Query fever (Q-fever) is a zoonotic disease that occurs globally, except in New Zealand [13], and it is caused by the obligate intercellular gram-negative bacterium *Coxiella burnetii* [14]. *Coxiella burnetii* infects pets in addition to livestock, which is a latent source of infection to humans [7]. This zoonosis is extremely flexible, and the infection can progress from acute and asymptomatic disease to chronic, and meningoencephalitis can occur. Perinatal infection (cattle, sheep and goats) and bronchopneumonia (sheep) [15] have also been ascribed to this pathogen. Query fever is transmitted by a wide array of tick species carrying about 40% of natural infection and shedding a significant number of viable organisms in their feces [16]. Since *C*. *burnetii* can be transmitted via aerosol inhalation, it has been classified under category B as a bioterrorism agent by the Centers for Disease Control and Prevention and the National Institute of Allergy and Infectious Diseases [14,17,18,19].

Rickettsioses are febrile illnesses caused by intracellular bacteria of the genus *Rickettsia* [20]. This genus is classified into spotted fever group (SFG) rickettsiae, typhus group (TG) rickettsiae, *Rickettsia bellii* group and *R*. *canadensis* group [21]. The SFG contains three species which are of medical importance in sub-Saharan Africa and have been identified as *Rickettsia africae* (African tick-bite fever (ATBF)), *R*. *conorii* (Mediterranean spotted fever (MSF)) and *R*. *aeschlimannii* [22,23,24]. In SA, the tourism industry has been negatively affected by rickettsiosis, even though there are no reported cases among the indigenous people because this disease does not have clinical signs; many clinical cases (infection and illness) have been reported from tourists returning to their home countries after visiting nature game reserves in SA [25]. African tick-bite fever (ATBF) is caused by *Rickettsia africae* and is mainly transmitted by *Amblyomma* species which are principal vectors in southern Africa [3]. Domestic animals infected by ATBF show no clinical symptoms of the disease and are intermittently rickettsemic and can be a source of infection for tick species [26].

To date, there is no published scientific report on the presence of zoonotic pathogens transmitted by various tick species in Lesotho. This study aimed to identify zoonotic tick-borne pathogens of economic importance in ticks infesting domestic animals in Lesotho using molecular techniques.

## 2. Results

Tick species collected from Lesotho districts were identified morphologically. In total, there were nine tick species from four major genera, namely *Haemaphysalis*, *Hyalomma*, *Rhipicephalus* and *Otobius*. Tick species identified were *Haemaphysalis elliptica*, *Hyalomma rufipes*, *Hyalomma truncatum*, *Otobius megnini*, *Rhipicephalus appendiculatus*, *Rhipicephalus decoloratus*, *Rhipicephalus evertsi evertsi*, *Rhipicephalus glabroscutatum* and *Rhipicephalus microplus* (Table 1). The overall number of tick species collected from each domestic animal, the gender and life stage of each tick species was documented, although in the current study these parameters were not analysed. Specimens (*Ha*. *elliptica*, *Hy*. *truncatum,* and *R*. *glabroscutatum*) were stored in glycerol during sampling, therefore no molecular analysis was performed due to DNA degradation. These specimens were only used for morphological identification purposes. From the overall ticks collected, a total of 322 pooled DNA samples were subjected to PCR amplification to detect the presence of targeted zoonotic pathogens of economic importance.

In this study, a 37% (121/322) prevalence of zoonotic pathogens from tick species of domestic animals in districts of Lesotho is reported. Zoonotic species of economic importance were detected, namely *Anaplasma* spp., *C*. *burnetii* and *R*. *africae*. Of the 322 pooled tick DNA samples, 121 samples were PCR positive for *Anaplasma* spp, *C*. *burnetii* and *R*. *africae* (Table 2). The highest prevalence of zoonotic pathogens occurred for *Anaplasma* spp. (35%), followed by *R*. *africae* (2%) and the least prevalent was *C*. *burnetii* (1%) (Table 3). The highest overall infection rate was 52% for ticks collected from goats and the lowest one was 26% for ticks collected from cattle (Table 3).

### 2.1. Anaplasma *Species*

**Figure 1:**
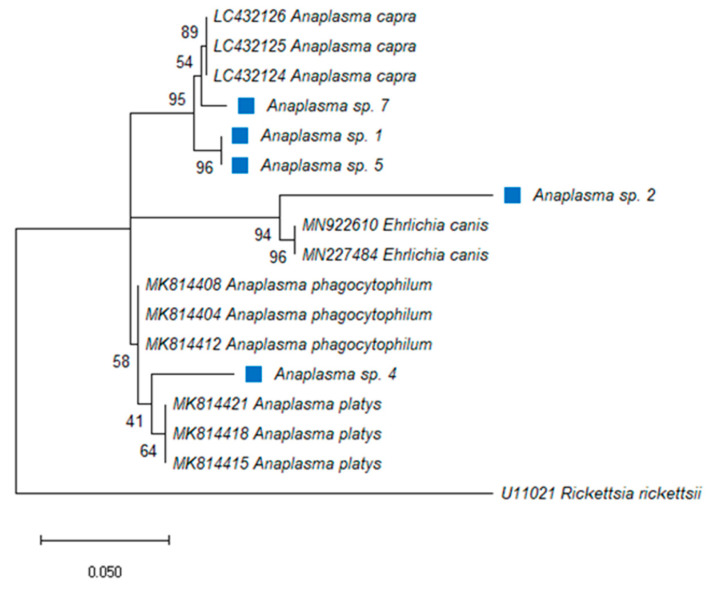
Phylogenetic analysis by Maximum Likelihood (ML) inferred from the 16S rRNA gene, method based on the Kimura 2-parameter model with gamma distributions (+G). The tree highlights the position of *Anaplasma* and *Ehrlichia* spp. The blue square indicates *Anaplasma* spp. identified in this study. Support values (bootstrap replicates of 1000) are indicated at each node of the branch.

For *Anaplasma* spp., 88% of the total samples tested positive and a corresponding band of 250 bp was detected by ethidium bromide agarose gel electrophoresis followed by UV illumination. The DNA of the pathogen was detected in the pools from seven districts, namely Berea, Butha-Buthe, Leribe, Maseru, Mohale’s Hoek, Qacha’s Nek and Quthing, whereas all the Mafeteng samples were PCR negative. In the Berea district, the infection rate with *Anaplasma* spp. was as high as 50% (Table 4). There was a significant difference in prevalence of *Anaplasma* spp. between tick species at *p* ≥ 0.103. The DNA of ticks identified as positive for the bacterium (*R*. *microplus*, *R*. *e*. *evertsi*, *Hy*. *rufipes*, *O*. *megnini* and *R*. *decoloratus*) via PCR were directly sequenced using a portion of the 16S rRNA gene. The sequences matched with uncultured *Anaplasma* species (MN481611; MN317257; MG241117) with identity ranging from 98–100% and coverage ranging from 92–99%. The phylogenetic analysis showed that *Anaplasma* spp. from Lesotho were closely related to other *Anaplasma* species, three samples were identified as *A*. *capra* (LC432124-26), two samples as *E*. *canis* (MN9222610; MN227484) and other samples matched with *A*. *platys* (MK814421; MK814418; MK814415) (Figure 1). Monophyletic relationship was observed in each clade. Most (51%) of the positive tick samples were from ticks infesting goats. There was a 100% infection rate of *Anaplasma* spp. detected solely in *R*. *microplus*, followed by *R*. *e*. *evertsi* with 92%. *Hyalomma rufipes* and *O*. *megnini* shared a 50% infection rate and *R*. *decoloratus* had a 40% prevalence.

### 2.2. Coxiella burnetii

The overall prevalence for *C*. *burnetii* was 1% (2/322). The infection rate in ticks collected from cattle was 4% (Table 2). Tick DNA from Butha-Buthe and Mohale’s Hoek district were PCR positive for the pathogen with an overall infection rate of 4% from cattle (Table 5). Of the positive samples, the highest infection rate was 20% in *R*. *decoloratus* and 1% in *R*. *e*. *evertsi*. The difference in *C*. *burnetii* prevalence between tick species was highly significant at *p* ≥ 0.998. Sequence analysis for the bacterium PCR positive sequenced samples revealed 96% maximum identity with *C*. *burnetii* MT268529-32 and MN025541.

**Figure 2:**
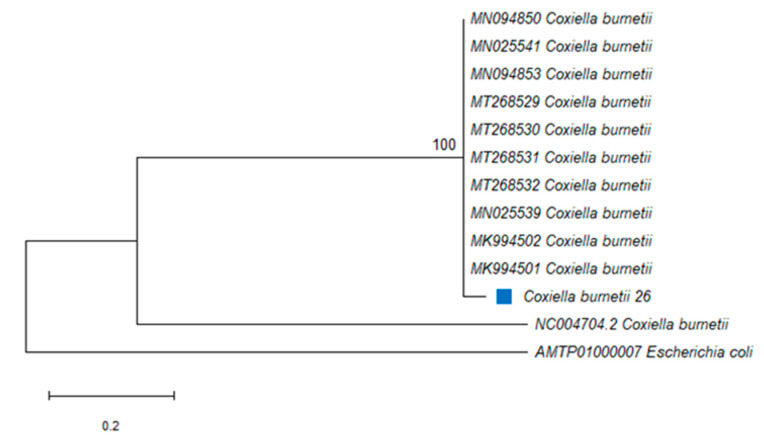
Phylogenetic analysis by Maximum Likelihood (ML) inferred from the IS1111 transposase gene, method based on the Kimura 2-parameter model. The blue square indicates *Coxiella burnetii* identified in this study. The tree highlights the position of *Coxiella burnetii*. Support values (bootstrap replicates of 1000) are indicated at each node of the branch.

The amplified PCR product of approximately 687 bp was obtained from *C*. *burnetii*-positive ticks. The PCR amplicons from the IS1111 transposase gene were generated from two ticks and sequenced, and DNA sequence analysis revealed that the sequence from *R*. *decoloratus* in Butha-Buthe and the other from *R*. *e*. *evertsi* in Mohale’s Hoek were identical. A *C*. *burnetii* sequence was identified from two tick species in this study and was submitted to GenBank and assigned as *C*. *burnetii* 26 (**MT592821**). The 493 nucleotide sequences of the IS1111 transposase gene from 11 bacterial species or isolates belonging to gammaproteobacterial were used to assess the phylogenetic relationships. A member of this class *Raoultella ornithinolytica* was used as an outgroup species. The phylogenetic analysis showed that *C*. *burnetii* from this study (designated *C*. *burnetii* 26) was closely related to *C*. *burnetii* (MT268531) isolated from a tick in Algeria and *C*. *burnetii* 26 formed a monophyletic group with other *C*. *burnetii* strains, with 100% identity (Figure 2).

### 2.3. Rickettsia africae

**Figure 3:**
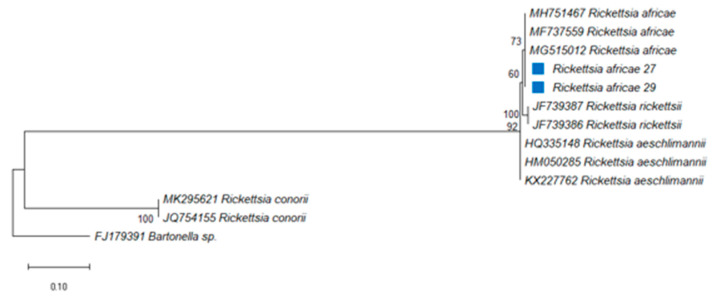
Phylogenetic analysis by Maximum Likelihood (ML) inferred from the *glt*A gene, method based on the Tamura 3-parameter model. The tree highlights the position of *Rickettsia africae*. The blue square indicates *Rickettsia africae* identified in this study. Support values (bootstrap replicates of 1000) are indicated at each node of the branch.

*Rickettsia africae* was present in ticks of domestic animals from Berea, Maseru, Qacha’s Nek and Quthing, but was absent in ticks collected from Butha-Buthe, Leribe, Mafeteng and Mohale’s Hoek (Table 6), having an overall 2% prevalence of infection. Quthing district had the highest infection rate of 40%. The highest overall infection rate was 22% in ticks collected from horses, followed by 2% in ticks from goats and, least shared, 1% prevalence was in ticks from cattle and sheep (Table 6). Tick species collected from the vegetation and donkeys were PCR-negative for the bacterium (Table 3). The difference in prevalence of *R*. *africae* between tick species was not significant at *p* ≥ 0.001. To confirm the pathogen identity, the *glt*A gene was sequenced revealing a 99% identity with *R*. *africae glt*A gene partial sequences for all the PCR positive samples (Accession number: MG515012, MH751467 and MH737559). Most of the positive samples (75%) were from *Hy*. *rufipes*, while 3% were detected in *R*. *e*. *evertsi*. The *O*. *megnini*, *R*. *appendiculatus*, *R*. *decoloratus* and *R*. *microplus* pools were negative for the presence of *R*. *africae*. The sequence of the amplified PCR product of the *R*. *africae glt*A gene was aligned and compared with other corresponding reference sequences in GenBank. The DNA sequences of *R*. *africae* from *R*. *e*. *evertsi* were submitted to GenBank and assigned as *R*. *africae* 27 (**MT585813**) and *R*. *africae* 29 (**MT585814**), respectively. The Maximum Likelihood (ML) phylogenetic tree of *R*. *africae* inferred from the partial sequence of the *glt*A gene indicated that *R*. *africae* 27 (MT585813) and *R*. *africae* 29 (MT585814) were 73% identical to other isolates of *R*. *africae* and were grouped together and closely related to *R*. *africae* (MH751467) from SA, *R*. *africae* (MF737559) from SA and *R*. *africae* (MG515012) from Brazil (Figure 3).

Coinfection was observed among zoonotic pathogens and with piroplasms that were detected in the same tick pools in a previous study, in ticks collected from cattle, goats, horses and vegetation (Table 7). Coinfection with *Anaplasma* spp. and *R*. *africae* were detected in *R*. *e*. *evertsi* and *Hy*. *rufipes* with rates of 1% and 11%, respectively. The coinfection rate ranged between 7–17% for *Anaplasma* spp. and *Babesia bigemina* in *R*. *e*. *evertsi* and *R*. *decoloratus,* respectively. Additionally, three pathogen coinfections were detected for *Anaplasma* spp., *B*. *motasi* and *B*. *ovis* in *R*. *e*. *evertsi*, with a 2% infection rate. The bivariate correlation test (data not shown) was not significant for the prevalence of zoonotic pathogens (*Anaplasma* spp. and *R*. *africae*) and those of piroplasms (*B*. *motasi* and *B*. *ovis*) coinfection (Table 7).

## 3. Discussion 

Control measures for most tick species is problematic as they live in close association with vertebrate hosts or in areas that are not easily accessible for acaricides application. Acaricides used to control vectors are not reliable. Currently, there are only two control methods used by farmers where tick species were collected: (1) For small stock, annual dipping, and government initiative campaigns on other injectable antiparasitic; (2) Injectable/pour-on antiparasitic, hand picking and rotational grazing for all the species.

As the first scientific publication on tick-borne pathogens, this study aimed to ascertain by molecular analysis the prevalence of tick-borne microorganisms of zoonotic importance in Lesotho. Species-specific PCR assay used were positive for pathogens detected from various tick species collected from domestic animals. Pathogens of economic importance, namely *Anaplasma* spp., *C*. *burnetii* and *R*. *africae,* were identified in ticks of domestic animals and vegetation in Lesotho. Furthermore, zoonotic bacteria, namely *Anaplasma* spp., *C*. *burnetii* and *R*. *africae* detected from *R*. *decoloratus* and *R*. *e*. *evertsi,* were confirmed through phylogenetic analysis, respectively.

In SA, *Hy*. *rufipes*, *R*. *decoloratus*, *R*. *e*. *evertsi* and *R*. *microplus* are common tick species with high prevalence and distribution [27,28]. *O*. *megnini* species have also been collected from cattle in the Eastern Cape, SA [29]. *Hyalomma rufipes* is a vector of the Crimean-Congo haemorrhagic fever virus [28] and *R*. *decoloratus* has been reported as a vector of *Babesia bigemina* in SA, which causes African redwater and *Anaplasma marginale*, a causative agent of gall sickness in cattle [30,31,32]. This tick is considered as one of the significant indigenous tick species harbored by cattle [31], while *R*. *microplus* is a competent vector of both *B*. *bigemina* and *B*. *bovis*, a causative agent of Asiatic redwater which is more virulent [30,32]. *Rhipicephalus e*. *evertsi* transmits *B*. *caballi* and *Theileria equi*, causative agents of equine piroplasmosis, and it also transmits *A*. *marginale* [27]. Thus far, *O*. *megnini* is not known to transmit any pathogen [33].

*Anaplasma* spp. infections were detected from tick species collected from cattle (22%) horses (22%), goats (51%), sheep (30%) and vegetation (33%). This pathogen has been detected in SA from ticks collected from dogs. This pathogen was identified and characterised through microscopic analysis of a blood sample from a dog in Bloemfontein, SA, by Inokuma [12], but the phylogenetic analysis showed that the pathogen differed significantly from *A*. *phagocytophilum* [34]. When compared to data collected in SA, the infection rate by this pathogen was lower, 17% in goats, 6% in cattle and 1.25% in sheep [5]. These findings indicate a clear existence of *Anaplasma* spp. in Lesotho with a higher than previously reported infection rate among the ticks infesting domestic animals.

Although we report the presence of *Anaplasma* spp. in ticks from domestic animals, further genetic characterisation needs to be conducted as sequences obtained in this study matched with uncultured *Anaplasma* species from the blood of cattle. The 16S rRNA gene used in this study did not provide sufficient findings in confirming the true existence of *A*. *phagocytophilum*; the phylogenetic analysis showed that the pathogen detected was from the genus *Anaplasma*. However, the species varied significantly. The obtained results do provide a baseline for the presence of *Anaplasma* pathogens in Lesotho and more studies must be conducted in the future to identify species of the genus *Anaplasma* that occur in this country using other assays. According to Mtshali [7], *Ixodes* species are vectors known to transmit this pathogen to humans causing HGA, but this vector is absent in SA which explains the lack of reported cases of this disease. Therefore, *Hy*. *rufipes*, *R*. *decoloratus*, *R*. *e*. *evertsi* and *R*. *microplus* should be considered as vectors that transmit *Anaplasma* spp. in Lesotho. Notably, *O*. *megnini* was also PCR positive for *Anaplasma* spp. and this is the first report to document likely transmission of the pathogen to a host by this soft tick. *Otobius megnini* adult soft ticks do not feed; only the larvae and nymphal developmental stages are considered to be parasitic [33]. Therefore, more investigation should be conducted in understanding the role of this tick in transmitting or harbouring pathogens. 

Interestingly, double, and triple bacterial coinfections were detected in ticks from domestic animals with *Anaplasma* spp. as a common bacterium in all multiple infections. The coinfection of zoonotic pathogens was detected from *Hy*. *rufipes* and *R*. *e*. *evertsi* collected from Quthing horses and Berea goats, respectively. These tick species were infected with *R*. *africae* and *Anaplasma* spp., although the infection rate was minimal. Piroplasms and zoonotic bacteria coinfections were detected in *R*. *e*. *evertsi* from Maseru ticks collected from cattle and vegetation, and Mafeteng ticks infesting on cattle were infected with a piroplasm parasite, *Babesia bigemina*. Additionally, concomitant infections were observed with *Anaplasma* spp., *B*. *bigemina* and *B*. *ovis* in ticks from Leribe goats. However, the rate of infection with three different parasites was minimal in *R*. *e*. *evertsi*. 

*Coxiella burnetii* is well known and has been reported throughout the African continent [35]. In West African countries, this pathogen has been identified through serology reports from *Amblyomma variegatum*, *Rhipicephalus senegalensis* and *Hyalomma truncatum* [7,36]. In SA, the infection caused by *C*. *burnetii* has been significantly demonstrated solely based on serological methods in cattle, goats and sheep, but recently Mtshali [5] identified the presence of *C*. *burnetii* from tick species infesting domestic animals in SA (Free State and KwaZulu-Natal) using molecular methods.

In this study we report a very low overall infection rate of 4% in ticks collected from cattle, while ticks collected from goats and sheep had no infection. These findings are corroborated by data from Mtshali [5] where the low incidence of 3% from cattle ticks, 6% from goats and 32% from sheep in SA was observed. In the early 1950s, Gummow [37] claimed that Q-fever levels are kept below a certain threshold within domestic animals, in particular cattle, in SA due to the low incidence considering the *C*. *burnetii* endemic stability. However, the bacterium incidence is still not well established and is still underestimated [7]. Additionally, Qiu [38] detected Q-fever and the infection rate was 7.8% for the overall ticks infesting domestic animals in Zambia; these findings are in line with this current study. Although the infection rates in ticks were low in tick species, this pathogen occurs in Lesotho. It can be ascertained if blood samples are studied.

Eight percent of *C*. *burnetii* seroprevalence from cattle has been reported in Transvaal, SA [39]. One human case was reported in Cape Town, where *C*. *burnetii* was found to be a cause of pneumonia in the 92-patient cohort [39]. No human reports have been documented recently in SA, suggesting that zoonotic pathogens are not well studied. Additionally, there are limited studies conducted on tick species that can be potential vectors transmitting zoonotic pathogens. Pets have been occasionally reported as the source of human infection of Q-fever as the pathogen can be easily shed by domestic animals. The environment can be contaminated from urine and faeces excreted by infected animals [5]. Therefore, due to a multitude of sources, the potential for human acquisition (including tick bites) are high [7]. In SA to date, the relationship between the bacteria and their pathogenicity to humans, and their prevalence, is unknown [7,40].

In this study, *C*. *burnetii* was identified for the first time in *R*. *decoloratus* collected from cattle, although *C*. *burnetii* has been reported from domestic animals in SA but not from vegetation [5], as in this study. Sequence analysis of the IS1111 gene from the genotype of *C*. *burnetii* detected in ticks revealed a close relationship to *C*. *burnetii* (MT268532), identified from (*Hyalomma dromedarii*) in Algeria, Africa. *Coxiella burnetii* identified in this study from *R*. *e*. *evertsi* was closely related to *C*. *burnetii* (MT268532; *Hy*. *dromedarii*), sharing at least 96.75%. The phylogenetic analysis confirms the bacterium identified as *C*. *burnetii*, a causative agent of human infection. If this pathogen is identified from a tick, as in this study, it can likely occur in humans through tick bites rather than from an infected animal shedding the bacteria. 

In sub-Saharan Africa and in rural settings, *R*. *africae* is transmitted by *Amblyomma hebraeum* [41]. Seroprevalence of ATBF (70%) has been documented in areas where *A*. *hebraeum* and cattle coincide. In Mpumalanga, SA, 24.5% of serological evidence (IgM antibodies) of ATBF in patients with febrile illness has been documented while, within the same community, 92.2% of patients were seropositive showing common exposure to ATBF rickettsiae [41]. Other cases reported on ATBF have been from people who visited SA as tourists [42]. Two percent of *R*. *africae* overall infection prevalence was observed in this study from four districts, namely Maseru, Berea, Qacha’s Nek and Quthing. In this investigation, the bacterium was isolated from *R*. *e*. *evertsi* and *Hy*. *rufipes* collected from cattle, goats, sheep, and horses. These tick species are well distributed in SA [27,28], but the knowledge of biological transmission of *R*. *africae* to humans is unknown while *A*. *hebraeum* is well documented, with 75% potential in transmitting the pathogen [7]. In this study, *Amblyomma* species were not collected, and based on these findings, it is likely that animals were intermittently rickettsiemic, so tick infection can be passive other than of the known vector, especially in rural areas where there is close contact between humans and animals. 

The sequence analysis of the *glt*A gene revealed that ticks from Lesotho domestic animals were infected with *R*. *africae*. To our knowledge, this is the first report in which *R*. *africae* was detected in *R*. *e*. *evertsi* and *Hy*. *rufipes* in Lesotho and the first report of *R*. *africae* infection rate in the country. The ML phylogenetic analysis showed that *R*. *africae* 27 (MT585813) and *R*. *africae* 29 (MT585814) both identified in *R*. *e*. *evertsi* grouped with *R*. *africae* (MG515012; MF737559; MH751467) from Brazil and two from SA, respectively, confirming that the bacteria isolated was indeed *R*. *africae*. The results also revealed that *R*. *africae* in this study were most closely related to *R*. *rickettsii*, a sister clade that is recognised as a zoonotic pathogen in SFG rickettsiae. The pathogenicity of *R*. *africae* isolated from *R*. *e*. *evertsi* from Lesotho should be studied further to map the prevalence of this zoonotic disease in domestic animals through blood samples. 

Interestingly, in this study, the SFG rickettsiae identified in *R*. *e*. *evertsi* that were collected from cattle and goats were genetically similar to *R*. *africae* (MG515012; MF737559) identified from human skin in Brazil and SA, respectively [43]. The strain isolated from Brazil was originally from a tourist who visited SA. Sequence analysis of the *R*. *africae glt*A gene isolated from ticks in this study indicates that the sequences shared 100% identity. These findings suggest that *R*. *e*. *evertsi* can serve as a vector for *R*. *africae* to humans. *Rhipicephalus* species role in pathogen transmission is not yet clear, and their infection may depend on coincidental transmission by other recognised vectors such as *A*. *hebraeum* [44].

*Rhipicephalus* species can be considered potential vectors for many tick-borne pathogens, including bacteria and protozoa [45]. However, more robust studies need to be conducted to confirm such and to further understand the mechanism and lifecycle of the pathogen. 

This study we recorded two confirmed ticks, namely *R*. *decoloratus* and *R*. *e*. *evertsi,* that were infected with *C*. *burnetii* and *R*. *africae* zoonotic pathogens and *Anaplasma* spp. pathogens detected from *Hy*. *rufipes*, *R*. *decoloratus*, *R*, *e*. *evertsi*, *R*. *microplus* and *O*. *megnini*. More robust genetic studies are needed to have better insight into the epidemiology of *Anaplasma* spp., *C*. *burnetii* and *R*. *africae* infections in tick species of Lesotho and the mode of transmission on domestic animals. Furthermore, other tick species must be considered as possible ideal vectors of zoonotic pathogens in the absence of well-documented vectors. The use of blood samples to confirm the infection of domestic animals in Lesotho is necessary and may be considered especially for the *Anaplasma* spp. with the use of more assays.

## 4. Materials and Methods

### 4.1. Tick Samples and Identification

**Figure 4:**
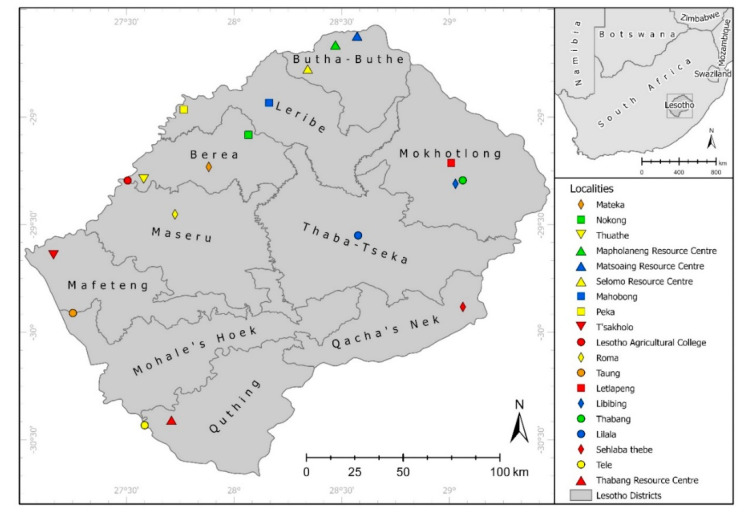
Map of Lesotho. Study area including the ten districts where sampling was carried out.

A total of 3311 individual ticks were collected during hot and wet seasons from December 2016 to May 2019 from domestic animals and vegetation. The specimens were collected from ten Lesotho districts, namely Berea, Butha-Buthe, Leribe, Mafeteng, Maseru Mohale’s Hoek, Mokhotlong, Qacha’s Nek, Quthing and Thaba Tseka (Figure 4). 

A total of 1683 ticks from the overall individuals collected were used for molecular analysis. Ticks were collected randomly from cattle, donkey, horses, goats, and sheep (Table 8), focusing on body parts that were severely infested (ears, neck, abdominal and perineum areas). Sterile fine-tipped forceps were used to detach the tick from the host. Questing ticks were collected on vegetation from inactive animal trails (Supplementary Table S1). Specimens were preserved in 70% (*v/v*) ethanol and subsequently identified morphologically using taxonomic keys Latif [46], Madder [47] and Walker [48]. Thereafter, representatives of each identified species were confirmed by a tick taxonomist at the Onderstepoort Veterinary Institute. The developmental stage and the sex of each tick species was recorded for each district sampled (Table 9).

### 4.2. DNA Extraction from Ticks

Ticks were pooled (one to four specimens) with specificity to species sex, life stage, host, vegetation and district. All specimens were surface sterilised twice with 70% ethanol and washed twice with sterile water, ensuring that all debris and animal hairs were removed before being crushed in 1.5 mL Eppendorf^®^ tubes. The genomic DNA (gDNA) was extracted using the Zymo tissue DNA extraction kit following the manufacturer’s protocol (Zymo Research Corporation, Irvine, CA, USA) and stored at −20 °C until used. 

### 4.3. Detection of Zoonotic Pathogens DNA by PCR

The pooled tick DNA was subjected to PCR amplification using oligonucleotide sequences listed in Table 10. The samples were screened for the presence of *A*. *phagocytophilum*, *C*. *burnetii* and *R*. *africae*. Positive controls for *C*. *burnetii* and *R*. *africae* were obtained from the Research Center for Zoonosis Control (CZC), Hokkaido University, Japan, and from the School of Medicine, Johns Hopkins University, Maryland. The positive control of *A. phagocytophilum* was obtained from blood collected from a Northern Cape horse in a study by Mlangeni [49]. For all reactions, ddH_2_O was used as a negative control. The infection rate was analysed by PCR methods using species-specific primers to detect zoonotic tick-borne pathogens. Coinfection among zoonotic pathogens and other parasites [50] was tested. The PCR reactions were performed in a 25 µL volume with 12.5 µL DreamTaq PCR Master Mix (Thermo Fisher Scientific, South Africa), 3 µL of each primer (10 µM each primer), 3 µL of genomic DNA template and 3.5 µL deionised water (ddH_2_O) was added to the final volume. All PCR reactions were performed using BIO-RAD T100 thermocycler (BIO-RAD Laboratories, South Africa). PCR conditions were as follows: pre-cycle denaturation at 95 °C for 10 min, followed by 35 cycles at 95 °C for 30 s, annealing (Table 10) for 30 s, extension at 72 °C for 1 min and 30 s and a final extension at 72 °C for 7 min. PCR product detection was conducted on a 1.5% agarose gels stained with ethidium bromide and visualised under UV transilluminator electrophoresis, ChemiDoc system (Biotechnology Laboratory, Neiker, Spain). 

### 4.4. Sequencing, Basic Local Alignment Search Tool (BLAST) and Phylogenetic Analysis 

The PCR amplicons were sent to (Inqaba Biotech, Pretoria, South Africa) for purification and sequencing. To confirm sequences obtained from all PCR analysis, nucleotide BLAST (BLASTn) was used (www.ncbi.nlm.nih.gov/blast/, accessed on 16 May 2020). The gene sequences with 80–100% similarity match scores were considered as significant. The gene sequences were aligned using Clustal W, using multiple alignments under default parameters in MEGA X software [54]. Thereafter, the aligned sequences were trimmed to remove uneven ends from the aligned sequences. The trimmed alignment was subsequently transferred to MEGA X for Maximum Likelihood (ML) analyses. Phylogenetic analysis was used to interrogate the relationship between the sequences from pathogens identified in this study with other pathogen sequences (reference sequences obtained from GenBank) and to confirm the identities of our pathogen sequences.

### 4.5. Statistical Analyses 

The prevalence of each pathogen species was represented and summarised in tables. A descriptive test was used to determine the difference at *p* ≥ 0.05 (Pearson Chi-square value, asymptotic significance 2-sided) for the prevalence of *Anaplasma* spp, *C*. *burnetii* and *R*. *africae* zoonotic pathogens among tick species. The regression test was used to test if the presence of the pathogen is dependent on tick species using binary logistic statistical analysis. Thereafter, a correlation test was conducted to determine the influence of pathogens on each other using bivariate statistical test. The IBM SPSS software (Version 27, 2020) was used for the analyses. 

## Figures and Tables

**Table 1:** Abundance and prevalence (%) of tick species identified in ten districts from Lesotho.

Tick Species	Number of Ticks per District										Total No. of Ticks
	Berea (%)	Butha-Buthe (%)	Leribe (%)	Mafeteng (%)	Maseru (%)	Mohale’s Hoek (%)	Mokhotlong (%)	Qacha’s Nek (%)	Quthing (%)	Thaba Tseka (%)	
*Haemaphysalis elliptica*	0 (0)	0 (0)	0 (0)	0 (0)	0 (0)	0 (0)	0 (0)	2 (100)	0 (0)	0 (0)	2
*Hyalomma rufipes*	3 (3.4)	0 (0)	0 (0)	0 (0)	8 (9.2)	7 (8.0)	0 (0)	66 (75.9)	3 (3.4)	0 (0)	87
*Hyalomma truncatum*	0 (0)	0 (0)	13 (31.7)	0 (0)	6 (14.6)	0 (0)	0 (0)	22 (53.7)	0 (0)	0 (0)	41
*Otobius megnini*	0 (0)	0 (0)	0 (0)	38 (8.4)	48 (10.6)	76 (16.9)	0 (0)	289 (64.1)	0 (0)	0 (0)	451
*Rhipicephalus appendiculatus*	0 (0)	0 (0)	0 (0)	0 (0)	3 (100)	0 (0)	0 (0)	0 (0)	0 (0)	0 (0)	3
*Rhipicephalus decoloratus*	0 (0)	4 (1.3)	43 (14.0)	24 (7.8)	190 (61.7)	0 (0)	33 (10.7)	14 (4.5)	0 (0)	0 (0)	308
*Rhipicephalus e. evertsi*	161 (7.5)	168 (7.8)	647 (30.0)	13 (0.6)	244 (11.3)	190 (9.0)	27 (1.3)	694 (32.2)	5 (0.2)	7 (0.3)	2156
*Rhipicephalus glabroscutatus*	0 (0)	8 (18.6)	0 (0)	0 (0)	3 (7.0)	0 (0)	0 (0)	32 (74.4)	0 (0)	0 (0)	43
*Rhipicephalus microplus*	0 (0)	111 (52.7)	43 (19.5)	0 (0)	16 (7.3)	0 (0)	0 (0)	45 (20.5)	0 (0)	5 (2.3)	220
Total no. per district	164 (5.0)	291 (6.6)	746 (22.5)	75 (2.3)	518 (15.6)	273 (8.2)	60 (1.8)	1164 (35.2)	8 (0.2)	12 (0.4)	3311

**Table 2:** Tick species that tested PCR positive for zoonotic pathogens.

Ticks	Total No. of Tick Pools Screened (n)	*Anaplasma* spp.	*Coxiella burnetii*	*Rickettsia africae*	Total No. of (+ve) Tick Pools for Pathogens
*Hyalomma rufipes*	6	2	*-	3	5
*Otobius megnini*	8	1	-	-	1
*Rhipicephalus appendiculatus*	1	-	-	-	-
*Rhipicephalus decoloratus*	20	2	1	-	3
*Rhipicephalus e. evertsi*	280	106	1	3	110
*Rhipicephalus microplus*	7	2	-	-	2
Total no. of (+ve) tick samples	322	113	2	6	121

*- = negative for the pathogen; +ve = positive ticks for the pathogen.

**Table 3:** Overall infection rates of ticks collected from domestic animals and number of tested and positive tick pools with targeted zoonotic pathogens in Lesotho.

Hosts	Total No. of Tick Pools (n)	*Anaplasma* spp. (%)	*Coxiella burnetii* (%)	*Rickettsia africae* (%)	Total No. of Tick Species of Domestic Animals (%)
Cattle	73	16 (22)	2 (3)	1 (1)	19 (26)
Donkeys	5	*-	-	-	-
Goats	116	59 (51)	-	2 (2)	61 (52)
Horses	9	2 (22)	-	2 (22)	4 (44)
Sheep	113	34 (30)	-	1 (1)	35 (31)
Vegetation	6	2 (33)	-	-	2 (33)
Zoonotic pathogens overall infection rate (%)	322	113 (35)	2 (1)	6 (2)	121 (37)

*- = negative for the pathogen.

**Table 4:** Overall infection rates of *Anaplasma* spp. in ticks from domestic animals and vegetation in Lesotho districts.

Study Group	*Anaplasma* spp.	Berea	Butha-Buthe	Leribe	Maseru	Mohale’ s Hoek	Qacha’s Nek	Quthing	Total Screened (+ve) Samples per Host and Overall %
Cattle	Total tested	0	45	2	17	4	0	0	68
	No. of (+ve)	0	11	*-	5	-	0	0	16
	%	0	24	-	29	-	0	0	24
Goats	Total tested	16	1	50	0	4	45	0	116
	No. of (+ve)	10	1	30	0	-	18	0	59
	%	63	100	60	0	-	40	0	51
Sheep	Total tested	24	6	50	0	6	27	0	113
	No. of (+ve)	10	-	19	0	-	5	0	34
	%	42	-	38	0	-	19	0	30
Horses	Total tested	0	3	0	0	1	0	5	9
	No. of (+ve)	0	-	0	0	-	0	2	2
	%	0	-	0	0	-	0	40	22
Donkeys	Total tested	0	0	0	0	5	0	0	5
	No. of (+ve)	0	0	0	0	-	0	0	-
	%	0	0	0	0	-	0	0	-
Vegetation	Total tested	0	0	0	6	0	0	0	6
	No. of (+ve)	0	0	0	2	0	0	0	2
	%	0	0	0	33	0	0	0	33
Total screened		40	55	102	23	20	72	5	317
(+ve) samples and		20	12	49	7	-	23	2	113
overall % per district		50	22	48	30	-	32	40	36

+ve = positive samples, 0 = no samples collected, *- = negative for the pathogen.

**Table 5:** *Coxiella burnetii* overall infection rate (%) in ticks from cattle in Lesotho districts.

Sampling Site	Studied Animals	*Coxiella burnetii*		
District	Study Group	Positive (+ve)	Total Screened	Overall (%)
Butha-Buthe	Cattle	1	45	2
Mohale’s Hoek		1	4	25
Overall prevalence		2	49	4

+ve = positive samples.

**Table 6:** Overall infection rates of *Rickettsia africae* in ticks from domestic animals in Lesotho districts.

Study Group	*Rickettsia africae*	Berea	Butha-Buthe	Leribe	Maseru	Mohaleshoek	Qacha’s Nek	Quthing	Overall % (+ve) per Population
Cattle	Total tested	0	45	2	17	4	0	0	68
	No. of (+ve)	0	0	*-	1	-	0	0	1
	%	0	0	-	6	-	0	0	1
Goats	Total tested	16	1	50	0	4	45	0	116
	No. of (+ve)	1	-	-	0	-	1	0	2
	%	6	-	-	0	-	2	0	2
Sheep	Total tested	24	6	50	0	6	27	0	113
	No. of (+ve)	1	-	-	0	-	-	0	1
	%	4	-	-	0	-	-	0	1
Horses	Total tested	0	3	0	0	1	0	5	9
	No. of (+ve)	0	-	0	0	-	0	2	2
	%	0	-	0	0	-	0	40	22
Total screened		40	55	102	17	15	72	5	306
Overall (+ve) samples		2	-	-	1	-	1	2	6
Overall % per district		5	-	-	3	-	1	40	2

+ve = positive samples, 0 = no samples collected, *- = negative for the pathogen.

**Table 7:** Coinfection among zoonotic pathogens and other hemoparasites as reported in the literature.

Coinfections	Pathogens	Prevalence N (%)			
		Cattle	Goats	Horses	Vegetation
Two pathogens	*Anaplasma* spp. + *R*. *africae*	*-	1 (1%)	1 (11%)	
	*Anaplasma* spp. + *Babesia bigemina*	5 (7%)	-	-	1 (17%)
Three pathogens	*Anaplasma* spp. + *Babesia motasi* + *B*. *ovis*	-	2 (2%)	-	-

*- = no coinfection.

**Table 8:** Total number of tick species collected from domestic animals and vegetation in each sampled district in Lesotho. Quthing had the lowest samples.

Country	Districts											
Lesotho	Hosts	Berea	Butha-Buthe	Leribe	Mafeteng	Maseru	Mohale’s Hoek	Mokhotlong	Qacha’s Nek	Quthing	Thaba Tseka	Total per Host
	Cattle	*-	257	177	75	333	97	43	331	-	9	1322
	Dogs	-	-	3	-	-	-	-	70	-	-	73
	Donkeys	-	-	-	-	-	12	-	-	-	-	12
	Goats	67	18	219	-	-	15	7	141	-	3	470
	Horses	-	-	3	-	-	30	-	88	8	-	129
	Sheep	97	12	335	-	-	119	10	170	-	-	743
	Vegetation	-	4	9	-	185	-	-	364	-	-	562
Total per district		164	291	746	75	518	273	60	1164	8	12	3311

^∗^- =not collected.

**Table 9:** Sex and development stages of tick specimens identified for each district sampled.

Gender and Stage	Berea	Butha-Buthe	Leribe	Mafeteng	Maseru	Mohale’s Hoek	Mokhotlong	Qacha’s Nek	Quthing	Thaba Tseka	Total (Gender and Stage)
Male Adult	101	118	232	12	116	81	23	239	5	7	934
Female Adult	63	119	442	14	205	94	29	424	3	5	1398
Nymphs	0	54	72	49	116	98	8	501	0	0	898
Total (district)	164	291	746	75	518	273	60	1164	8	12	3311

**Table 10:** Oligonucleotide sequences used for amplification of *Anaplasma phagocytophilum*, *Coxiella burnetii* and *Rickettsia africae* targeted genes.

Pathogen	Target Genes	Primer Sequences	Product Size (bp)	Annealing Temp (°C)	Reference
*Anaplasma phagocytophilum*	16S rRNA	**EHR521F:** 5′-TGTAGGCGGTTCGGTAAGTTAAAG-3′ **EHR747R:** 5′-GCACTCATCGTTTACAGCGTG-3′	250	60	[51]
*Coxiella burnetii*	IS1111 transposase	**Trans1-F:** 5′-TATGTATCCACCGTAGCCAGTC-3′ **Trans2-R:** 5′-CCCAACAACACCTCCTTATTC-3′	687	60	[52]
*Rickettsia africae*	*glt*A	**CS-78:** 5′-GCAAGTATCGGTGAGGATGTAAT-3′ **CS-323:** 5′-GCTTCCTTAAAATTCAATAAATCAGGAT-3′	401	55	[53]

## Data Availability

Not applicable.

## Supplementary Materials

The following are available online at https://www.mdpi.com/article/10.3390/pathogens10091186/s1, Table S1: Tick species collected from domestic animals and districts sampled.
