# Supplementary material for: Molecular Detection of Coxiella burnetii, Rickettsia africae and Anaplasma Species in Ticks from Domestic Animals in Lesotho
Source: Pathogens. 2021 Sep 14;10(9):1186. doi: 10.3390/pathogens10091186 (PMC8468460; doi:10.3390/pathogens10091186)
Supplement: Supplementary file 1 [file pathogens-10-01186-s001.zip › pathogens-1312435-supplementary.pdf]

Table S1. Tick species collected from domestic animals and districts sampled.

**Lesotho districts**	**Studied animals**	**Tick species identified**								
		H. elliptica	*H. rufipes*	*H. truncatum*	*O. megnini*	*R. appendiculatus*	*R. decoloratus*	*R. microplus*	*R. e. evertsi*	*R. glabroscutatus*
**Berea (N=164)**	Goats	*-	-	-	-	-	-	-	67	-
	Sheep	-	3	-	-	-	-	-	94	-
**Butha-Buthe (N= 291)**	Cattle	-	-	-	-	-	3	95	151	8
	Goats	-	-	-	-	-	-	16	2	-
	Sheep	-	-	-	-	-	-	-	12	-
	Unknown host	-	-	-	-	-	1	-	3	-
**Leribe (N= 746)**	Cattle	-	-	4	-	-	42	40	91	-
	Dogs	-	-	-	-	-	-	3	-	-
	Goats	-	-	-	-	-	-	-	219	-
	Horses	-	-	-	-	-	-	-	3	-
	Sheep	-	-	9	-	-	1	-	325	-
	Unknown host	-	-	-	-	-	-	-	9	-
**Mafeteng (N= 75)**	Cattle	-	-	-	38	-	24	-	13	-
**Maseru (N= 518)**	Cattle	-	2	1	35	-	174	14	104	3
	Vegetation	-	-	-	-	3	2	-	59	-
	Unknown host	-	6	5	13	-	14	2	81	-
**Mohale's hoek (N= 273)**	Cattle	-	4	-	35	-	-	-	58	-
	Donkeys	-	-	-	-	-	-	-	12	-
	Goats	-	-	-	2	-	-	-	13	-
	Horses	-	3	-	10	-	-	-	17	-
	Sheep	-	-	-	29	-	-	-	90	-
**Mokhotlong (N= 60)**	Cattle	-	-	-	-	-	33	-	10	-
	Goats	-	-	-	-	-	-	-	7	-
	Sheep	-	-	-	-	-	-	-	10	-
**Qacha's Nek (N= 1164)**	Cattle	-	19	4	134	-	-	37	137	-
	Dogs	2	-	4	24	-	-	3	5	32
	Goats	-	1	-	1	-	-	-	139	-
	Horses	-	-	2	1	-	-	-	85	-
	Sheep	-	-	-	51	-	-	-	119	-
	Unknown host	-	46	12	78	-	14	5	209	-
**Quthing (N= 8)**	Horses	-	3	-	-	-	-	-	5	-
**Thaba Tseka (N= 12)**	Cattle	-	-	-	-	-	-	5	4	-
	Goats	-	-	-	-	-	-	-	3	-

*** - = not collected**
